# In This Issue

**DOI:** 10.1002/1097-0061(20000630)17:2<79::AID-YEA21>3.0.CO;2-X

**Published:** 2000


**Yeast**
*Yeast* 2000; **17**: 79–80.

### In this Issue

Proteomics is a rapidly growing area of functional genomics, and there is no doubt that Craig Venter's highly publicized, if rather late, entry into this field will add to the pace of technological advancement. His partners in this venture, PE Biosystems, plan to develop high speed mass spectrometers to be used to identify each individual protein from the mixtures obtained from cell or tissue samples.

### Proteome profiling — pitfalls and progress

In this review, Paul Haynes and John Yates examine the current state of analytical methods in proteomics, in which mass spectrometry is the common final step. To date, the most widely used approach has been to separate protein samples by 2D gel electrophoresis. However, this method does not allow the preservation of proteins as complexes. Alternative techniques have been developed to avoid this problem, allowing researchers to discover which proteins exist as complexes with other proteins; the advantages of these approaches as compared with 2D gel electrophoresis are discussed in the review.

Beyond the quantification of each protein present in the cell lies the determination of the interactions between proteins and their state of post-translational modification. In this issue, we highlight the development of the two-hybrid protein interaction screen into a genome-wide technique.

### Protein interaction mapping using the two-hybrid system

The role of global two-hybrid interaction screens in proteomics is discussed by Albertha Walhout, Simon Boulton and Marc Vidal. Two-hybrid screening is an established approach for identifying protein interaction partners. In this review, the advance into whole-genome 'global' two-hybrid screens is charted by discussion of the recent large-scale studies in *Saccharomyces cerevisiae* and *Caenorhabditis elegans.* The various approaches that have been used so far are compared with each other and the advantages and drawbacks of each are considered. Several other issues, such as how the data being generated by these approaches should be assessed and the average number of 'interaction sequence tags' per protein to be expected from such a screen, are also discussed.

### Global two-hybrid screen in yeast yields new splicing factors

Fromont-Racine *et al.* have used a genome wide two-hybrid screen with yeast genes encoding proteins thought to be involved in splicing as baits. The Sm proteins form the core of the snRNP particles that contain the spliceosomal snRNAs. A family of Sm-like (Lsm) proteins were previously detected by the authors in sequence homology searches of the yeast genome. These were used as baits in exhaustive two-hybrid screens with a mixture of whole and domain-encoding regions of all yeast genes as prey. In addition to detecting interactions with known splicing factors, they detected extensive interactions between these proteins, suggesting that they act as a complex. They have produced interaction maps for all of the genes tested and in some cases have defined which domains of the prey proteins interact with the bait.

The field of microbial genomics is benefiting greatly from the results of comparative genomics studies. The small genomes of these organisms make them popular targets for genome sequencing and a formidable collection of genomes is available for study. The comparison of genomes is of great interest to those studying pathogens, since comparisons to closely related non-pathogenic strains or to attenuated strains may be the way forward in determining which changes cause a strain to be pathogenic or determine immunogenicity.

### Comparative genomics of *Mycobacterium bovis* BCG Pasteur

Brosch *et al.* report on a comparison between the genomes of *Mycobacterium tuberculosis* and *Mycobacterium bovis* BCG Pasteur. This study has

Copyright © 2000 John Wiley & Sons, Ltd.

uncovered two major rearrangements in the *M. bovis* genome with respect to the *M. tuberculosis* genome. These are two tandem duplications, one of which is complete and one of which was subsequently partly deleted. The strains carrying these duplications are diploid for at least 58 genes and have two copies of the chromosomal origin of replication. As yet, the role of these duplications in the attenuation or altered immunogenicity of BCG is unknown, but these findings present interesting avenues for future investigation.

Microbial genomes were well represented at the Genomes 2000 meeting in Paris in April, with talks on human pathogens such as *Listeria monocytogenes* and *Mycobacterium leprae* and on classical model organisms such as *Bacillus subtilis* and *Escherichia coli.* We present a review of this meeting for those of our readers who were not fortunate enough to have the chance to sample 'Paris in springtime'.

### Meeting highlights—Genomes 2000

This meeting was organized jointly by the Institut Pasteur and the American Society for Microbiology (ASM). The meeting covered a wide range of organisms from mammalian systems, to plants, to microbes, the emphasis being on the latter category. There were sessions on functional genomics, computational genomics, structural genomics and evolution and comparative genomics. Presentations from all of these are detailed in this review of the meeting.

Mammalian comparative mapping is at a much less advanced stage, due to the incomplete nature of the genomes available for study. This is set to change, with Celera Genomics, USA, announcing the completion of its first draft of the human genome and 'officially' announcing the start of its mouse genome sequencing initiative. There are several resources on the web for mouse genome data. In this issue we review the largest and most established facility for mouse genomics.

### Website review—MGI

The Mouse Genome Informatics site, hosted by the Jackson Laboratory in Maine, USA, is an impressive collection of data on the mouse, from genome and gene data to expression data to literature citations and even tumour biology reports. There are several services for those interested in mammalian homology, which include Oxford grids and a map-drawing tool. The expression data can be searched with a chosen gene or region of interest and in some cases includes images of the data. The searches are fully supported by overviews and help links, making the site user friendly, and links back to other pages make navigation around the site quick and easy.

The release of the near-complete genome sequence of the fruit fly, *Drosophila melanogaster*, this March sent the usage statistics of sequencing databases sky-high in a frenzy of BLAST searching. This was the third eukaryotic genome to be completed and comprised some 120 Mb of sequence. *Drosophila melanogaster* is our featured organism for this issue, with a special 'how to' feature for those of you dying to get your hands on a fly mutant for your favourite gene.

### Featured organism—Drosophila

This 3 mm long fruit fly has long been the obsession of many researchers working on developmental and cellular pathways that this small dipteran shares with humans. This article gives non-fly researchers a background on the fly and discusses the approaches currently being applied to fly functional and comparative genomics. There is a listing of top fly websites and details on the current status of knowledge of the *Drosophila* genome. Steve Russell of the Genetics Department of Cambridge University, UK, and Rachel Drysdale of the Cambridge group of FlyBase share their views of where fly genomics will go next and their plans for the future.

The excitement over the completion of the fly genome was felt most strongly at the 41st Annual *Drosophila* Research Conference in Pittsburgh, USA, in fact the paper came out in *Science* during the meeting.

### Meeting review—41st Annual *Drosophila* Research Conference

Rachel Drysdale and Leyla Bayraktaroglu of Fly Base were at this momentous meeting and give us a report on the presentations given there and their views on the implications of the completion of the sequence.

Copyright © 2000 John Wiley & Sons, Ltd.

